# *catena*-Poly[[bis­(nitrato-κ*O*)copper(II)]-bis­[μ-1,4-bis­(pyridin-3-ylmeth­oxy)benzene-κ^2^ *N*:*N*′]]

**DOI:** 10.1107/S1600536811016096

**Published:** 2011-05-07

**Authors:** Ping Zou, Ying Liu, Guang-Feng Hou, Jin-Sheng Gao

**Affiliations:** a College of Life Science, Sichuan Agriculture University, Ya’an 625014, People’s Republic of China; b College of Chemistry and Materials Science, Heilongjiang University, Harbin 150080, People’s Republic of China; c Department of Materials and Chemistry Engineering, Heilongjiang Institute of Technology, Harbin 150050, People’s Republic of China

## Abstract

In the title compound, [Cu(NO_3_)_2_(C_18_H_16_N_2_O_2_)_2_]_*n*_, the Cu^II^ ion lies on an inversion center and is six-coordinated in a Jahn–Teller-distored octa­hedral geometry defined by four N atoms of the pyridine derivative forming a square plane, above and below which are the O atoms of the nitrate anion. The ligand links the metal atoms linto a linear chain running along the *a* axis. One of the nitrate O atoms is equally disordered over two sets of sites.

## Related literature

For the synthesis and background to network structures built up from flexible pyridyl-based aromatic ligands and transition metals, see Liu *et al.* (2010*a* ▶,*b* ▶).
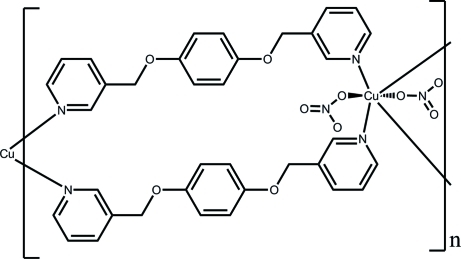


## Experimental

### 

#### Crystal data

[Cu(NO_3_)_2_(C_18_H_16_N_2_O_2_)_2_]
*M* *_r_* = 772.22
Monoclinic, 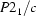

*a* = 8.4859 (17) Å
*b* = 17.030 (3) Å
*c* = 12.986 (4) Å
β = 116.22 (2)°
*V* = 1683.6 (7) Å^3^
*Z* = 2
Mo *K*α radiation
μ = 0.72 mm^−1^
*T* = 291 K
0.20 × 0.18 × 0.17 mm

#### Data collection

Rigaku R-AXIS RAPID diffractometer
Absorption correction: multi-scan (*ABSCOR*; Higashi, 1995 ▶) *T*_min_ = 0.867, *T*_max_ = 0.889
16256 measured reflections
3831 independent reflections
3067 reflections with *I* > 2σ(*I*)
*R*_int_ = 0.039

#### Refinement

*R*[*F* ^2^ > 2σ(*F* ^2^)] = 0.037
*wR*(*F* ^2^) = 0.097
*S* = 1.07
3831 reflections
251 parameters
12 restraints
H-atom parameters constrained
Δρ_max_ = 0.45 e Å^−3^
Δρ_min_ = −0.26 e Å^−3^

### 

Data collection: *RAPID-AUTO* (Rigaku, 1998 ▶); cell refinement: *RAPID-AUTO*; data reduction: *CrystalClear* (Rigaku/MSC, 2002 ▶); program(s) used to solve structure: *SHELXS97* (Sheldrick, 2008 ▶); program(s) used to refine structure: *SHELXL97* (Sheldrick, 2008 ▶); molecular graphics: *SHELXTL* (Sheldrick, 2008 ▶); software used to prepare material for publication: *SHELXL97*.

## Supplementary Material

Crystal structure: contains datablocks I, global. DOI: 10.1107/S1600536811016096/ng5152sup1.cif

Structure factors: contains datablocks I. DOI: 10.1107/S1600536811016096/ng5152Isup2.hkl

Additional supplementary materials:  crystallographic information; 3D view; checkCIF report

## Figures and Tables

**Fig. 1.**
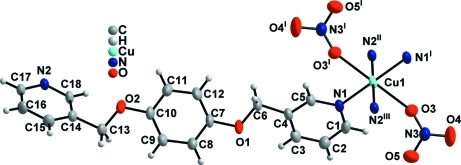
The molecular structure of the title compound, showing displacement ellipsoids at the 50% probability level for non-H atoms, disordered O4' atom has been omitted for clarity.

**Fig. 2.**
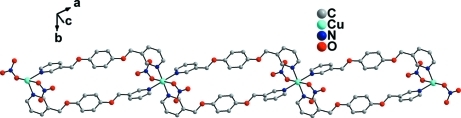
A partial packing view, showing the ribbon structure along [2 0 1] direction. Disordered O4' atoms and no involving H atoms have been omitted for clarity.

## supplementary crystallographic information

### Comment

The bridging pyridyl ligands can be used to construct interesting 0D to three-dimensional supramolecular architectures. Our group has reported some isolated molecule, chain, plane and three-dimensional network structures built up by flexible pyridyl-based aromatic ligands and transition metals. (Liu *et al.*, 2010*a*; Liu *et al.*, 2010*b*). As our continuing work for pyridyl ligands, we report here the synthesis and crystal structure of the title compound.

An asymmetric unit of the title compound consists of a 1,4-bis(pyridin-3-ylmethoxy)benzene molecule, a nitrate anion and a Cu^II^ cation (Figure 1). The Cu^II^ cations lie on the inversion centers and are six-coordinated in the Jahn-Teller distored octahedral geometry environments defined by four N atoms forming the square planes and two O atoms locating the polar axis.

In the crystal, ribbon structures along [2 0 1] direction are built up by heterocyclic ligands bridging Cu^II^ cations (Figure 2, Table 1).

### Experimental

The 1,4-bis(pyridin-3-ylmethoxy)benzene ligand was synthesized as the reference method (Liu *et al.*, 2010*a*): A mixture of 1,4-dihydroxybenzene (1.1 g, 10 mmol), 3-chloromethylpyridine hydrochloride (3.28 g, 20 mmol) and NaOH (1.6 g, 40 mmol) in acetonitrile (50 ml) was refluxed under nitrogen with stirring for 24 h. After cooling to room temperature, the solution was filtered and the residue was washed with acetonitrile for several times. The mixed filtrate was droped into 300 ml water solution to get the powder crude product. A total of 2.51 g (yield 86%) pure product was obtained by recrystallizing from the mixed solution of 10 ml water and 10 ml me thanol. The title compound was synthesized by reaction of 1,4-bis(pyridin-3-ylmethoxy)benzene ligand (0.29 g, 1.0 mmol) and Cu(NO_3_)_2_^.^3H_2_O (0.22 g, 1.0 mmol) in 5 ml water and 5 ml me thanol mixed solution. After filtration, blue block crystals suitable for X-ray diffraction were obtained by slow evaporation at room temperature for several days in 46% yield.

### Refinement

O4 atom of nitrate was disordered over two positions with site occupancy factors of *ca* 0.51 and 0.49, and then, the two positions were restraint refined with commond 'Iosr 0.01 O4 O4' '. H atoms bound to C atoms were placed in calculated positions and treated as riding on their parent atoms, with C—H = 0.93 Å (aromatic); C—H = 0.97 Å (methylene), and with *U*_iso_(H) = 1.2*U*_eq_(C).

### Crystal data

**Table:**

[Cu(NO_3_)_2_(C_18_H_16_N_2_O_2_)_2_]	*F*(000) = 798
*M**_r_* = 772.22	*D*_x_ = 1.523 Mg m^−^^3^
Monoclinic, *P*2_1_/*c*	Mo *K*α radiation, λ = 0.71073 Å
Hall symbol: -P 2ybc	Cell parameters from 13022 reflections
*a* = 8.4859 (17) Å	θ = 3.4–27.4°
*b* = 17.030 (3) Å	µ = 0.72 mm^−^^1^
*c* = 12.986 (4) Å	*T* = 291 K
β = 116.22 (2)°	Block, blue
*V* = 1683.6 (7) Å^3^	0.20 × 0.18 × 0.17 mm
*Z* = 2	

### Data collection

**Table:**

Rigaku R-AXIS RAPID diffractometer	3831 independent reflections
Radiation source: fine-focus sealed tube	3067 reflections with *I* > 2σ(*I*)
graphite	*R*_int_ = 0.039
ω scans	θ_max_ = 27.5°, θ_min_ = 3.4°
Absorption correction: multi-scan (*ABSCOR*; Higashi, 1995)	*h* = −11→10
*T*_min_ = 0.867, *T*_max_ = 0.889	*k* = −22→22
16256 measured reflections	*l* = −16→16

### Refinement

**Table:**

Refinement on *F*^2^	Primary atom site location: structure-invariant direct methods
Least-squares matrix: full	Secondary atom site location: difference Fourier map
*R*[*F*^2^ > 2σ(*F*^2^)] = 0.037	Hydrogen site location: inferred from neighbouring sites
*wR*(*F*^2^) = 0.097	H-atom parameters constrained
*S* = 1.07	*w* = 1/[σ^2^(*F*_o_^2^) + (0.0495*P*)^2^ + 0.4137*P*] where *P* = (*F*_o_^2^ + 2*F*_c_^2^)/3
3831 reflections	(Δ/σ)_max_ < 0.001
251 parameters	Δρ_max_ = 0.45 e Å^−^^3^
12 restraints	Δρ_min_ = −0.26 e Å^−^^3^

### Special details

**Table:**

Geometry. All e.s.d.'s (except the e.s.d. in the dihedral angle between two l.s. planes) are estimated using the full covariance matrix. The cell e.s.d.'s are taken into account individually in the estimation of e.s.d.'s in distances, angles and torsion angles; correlations between e.s.d.'s in cell parameters are only used when they are defined by crystal symmetry. An approximate (isotropic) treatment of cell e.s.d.'s is used for estimating e.s.d.'s involving l.s. planes.
Refinement. Refinement of *F*^2^ against ALL reflections. The weighted *R*-factor *wR* and goodness of fit *S* are based on *F*^2^, conventional *R*-factors *R* are based on *F*, with *F* set to zero for negative *F*^2^. The threshold expression of *F*^2^ > σ(*F*^2^) is used only for calculating *R*-factors(gt) *etc*. and is not relevant to the choice of reflections for refinement. *R*-factors based on *F*^2^ are statistically about twice as large as those based on *F*, and *R*- factors based on ALL data will be even larger.

### Fractional atomic coordinates and isotropic or equivalent isotropic displacement parameters (Å^2^)

**Table:**

	*x*	*y*	*z*	*U*_iso_*/*U*_eq_	Occ. (<1)
C1	−0.3511 (2)	0.87028 (12)	0.16557 (18)	0.0353 (4)	
H1	−0.4698	0.8612	0.1432	0.042*	
C2	−0.2290 (3)	0.82377 (13)	0.25037 (19)	0.0383 (5)	
H2	−0.2657	0.7837	0.2833	0.046*	
C3	−0.0520 (3)	0.83705 (12)	0.28601 (18)	0.0351 (4)	
H3	0.0317	0.8065	0.3435	0.042*	
C4	−0.0017 (2)	0.89662 (11)	0.23463 (17)	0.0303 (4)	
C5	−0.1310 (2)	0.93984 (12)	0.14835 (18)	0.0325 (4)	
H5	−0.0970	0.9788	0.1120	0.039*	
C6	0.1879 (2)	0.91934 (13)	0.27306 (19)	0.0385 (5)	
H6A	0.2070	0.9342	0.2073	0.046*	
H6B	0.2181	0.9637	0.3252	0.046*	
C7	0.4713 (2)	0.86583 (12)	0.39043 (19)	0.0372 (5)	
C8	0.5671 (2)	0.80017 (12)	0.44675 (18)	0.0350 (4)	
H8	0.5101	0.7526	0.4415	0.042*	
C9	0.7480 (2)	0.80513 (12)	0.51103 (18)	0.0349 (4)	
H9	0.8124	0.7609	0.5482	0.042*	
C10	0.8320 (2)	0.87615 (12)	0.51957 (18)	0.0360 (5)	
C11	0.7367 (3)	0.94163 (13)	0.4634 (2)	0.0419 (5)	
H11	0.7937	0.9892	0.4690	0.050*	
C12	0.5558 (3)	0.93666 (13)	0.3987 (2)	0.0425 (5)	
H12	0.4917	0.9808	0.3611	0.051*	
C13	1.1170 (2)	0.82248 (12)	0.63460 (19)	0.0381 (5)	
H13A	1.1171	0.7852	0.5781	0.046*	
H13B	1.0739	0.7963	0.6835	0.046*	
C14	1.2990 (2)	0.85426 (11)	0.70499 (17)	0.0305 (4)	
C15	1.4412 (3)	0.83865 (12)	0.68279 (19)	0.0375 (5)	
H15	1.4282	0.8078	0.6205	0.045*	
C16	1.6027 (3)	0.86992 (12)	0.75514 (19)	0.0377 (5)	
H16	1.6998	0.8600	0.7418	0.045*	
C17	1.6204 (2)	0.91564 (11)	0.84668 (18)	0.0319 (4)	
H17	1.7306	0.9354	0.8955	0.038*	
C18	1.3260 (2)	0.90220 (11)	0.79731 (16)	0.0300 (4)	
H18	1.2301	0.9139	0.8113	0.036*	
Cu1	−0.5000	1.0000	0.0000	0.03106 (12)	
N1	−0.30485 (19)	0.92812 (9)	0.11430 (14)	0.0304 (3)	
N2	1.48165 (18)	0.93268 (9)	0.86752 (13)	0.0279 (3)	
N3	−0.8293 (2)	0.86505 (11)	−0.01848 (17)	0.0411 (4)	
O1	0.29285 (18)	0.85382 (9)	0.32890 (17)	0.0579 (5)	
O2	1.01067 (18)	0.88797 (9)	0.57993 (16)	0.0536 (5)	
O3	−0.74035 (19)	0.92569 (8)	0.02595 (14)	0.0421 (4)	
O4	−0.9828 (11)	0.8623 (6)	−0.035 (2)	0.085 (4)	0.49 (4)
O5	−0.7554 (2)	0.80297 (10)	−0.01836 (19)	0.0654 (5)	
O4'	−0.9848 (9)	0.8748 (6)	−0.0923 (18)	0.080 (4)	0.51 (4)

### Atomic displacement parameters (Å^2^)

**Table:**

	*U*^11^	*U*^22^	*U*^33^	*U*^12^	*U*^13^	*U*^23^
C1	0.0205 (9)	0.0423 (11)	0.0386 (11)	−0.0021 (8)	0.0088 (8)	−0.0068 (9)
C2	0.0323 (10)	0.0405 (11)	0.0392 (12)	−0.0036 (9)	0.0132 (9)	0.0031 (9)
C3	0.0270 (9)	0.0401 (11)	0.0304 (10)	0.0059 (8)	0.0056 (8)	0.0042 (8)
C4	0.0195 (8)	0.0351 (10)	0.0305 (10)	0.0041 (7)	0.0058 (7)	−0.0003 (8)
C5	0.0216 (9)	0.0352 (10)	0.0343 (10)	0.0040 (7)	0.0065 (8)	0.0048 (8)
C6	0.0194 (9)	0.0427 (11)	0.0436 (12)	0.0033 (8)	0.0049 (9)	0.0093 (9)
C7	0.0152 (8)	0.0442 (11)	0.0429 (12)	0.0024 (8)	0.0044 (8)	0.0069 (9)
C8	0.0236 (9)	0.0351 (10)	0.0415 (12)	−0.0008 (8)	0.0100 (9)	0.0045 (9)
C9	0.0233 (9)	0.0365 (10)	0.0360 (11)	0.0040 (8)	0.0050 (8)	0.0043 (8)
C10	0.0179 (8)	0.0424 (11)	0.0355 (11)	−0.0001 (8)	0.0008 (8)	0.0011 (9)
C11	0.0252 (10)	0.0369 (11)	0.0524 (14)	−0.0033 (8)	0.0069 (9)	0.0042 (10)
C12	0.0247 (10)	0.0383 (11)	0.0518 (13)	0.0057 (8)	0.0054 (9)	0.0119 (10)
C13	0.0216 (9)	0.0371 (10)	0.0417 (12)	0.0011 (8)	0.0012 (8)	−0.0056 (9)
C14	0.0206 (8)	0.0306 (9)	0.0308 (10)	0.0011 (7)	0.0028 (8)	−0.0006 (8)
C15	0.0323 (10)	0.0383 (11)	0.0383 (11)	0.0028 (8)	0.0123 (9)	−0.0087 (9)
C16	0.0251 (9)	0.0400 (11)	0.0489 (13)	0.0013 (8)	0.0172 (9)	−0.0063 (9)
C17	0.0184 (8)	0.0316 (9)	0.0385 (11)	0.0010 (7)	0.0059 (8)	−0.0019 (8)
C18	0.0164 (8)	0.0376 (10)	0.0299 (10)	0.0025 (7)	0.0046 (7)	−0.0007 (8)
Cu1	0.01777 (16)	0.03750 (19)	0.02666 (18)	0.00767 (13)	−0.00042 (12)	−0.00511 (14)
N1	0.0193 (7)	0.0353 (8)	0.0292 (8)	0.0048 (6)	0.0041 (6)	−0.0024 (7)
N2	0.0186 (7)	0.0312 (8)	0.0289 (8)	0.0032 (6)	0.0059 (6)	−0.0011 (6)
N3	0.0276 (9)	0.0427 (10)	0.0492 (11)	−0.0003 (7)	0.0136 (8)	0.0019 (8)
O1	0.0167 (7)	0.0448 (9)	0.0878 (14)	0.0030 (6)	0.0009 (8)	0.0187 (9)
O2	0.0205 (7)	0.0427 (9)	0.0697 (12)	−0.0023 (6)	−0.0055 (7)	0.0095 (8)
O3	0.0392 (8)	0.0356 (7)	0.0475 (9)	−0.0028 (6)	0.0154 (7)	−0.0007 (7)
O4	0.028 (2)	0.096 (4)	0.124 (9)	0.000 (2)	0.027 (4)	0.004 (5)
O5	0.0593 (11)	0.0374 (9)	0.0899 (15)	0.0023 (8)	0.0243 (11)	−0.0022 (9)
O4'	0.022 (2)	0.086 (4)	0.102 (8)	−0.005 (2)	0.001 (3)	−0.006 (4)

### Geometric parameters (Å, °)

**Table:**

C1—N1	1.341 (3)	C13—O2	1.413 (2)
C1—C2	1.380 (3)	C13—C14	1.505 (3)
C1—H1	0.9300	C13—H13A	0.9700
C2—C3	1.381 (3)	C13—H13B	0.9700
C2—H2	0.9300	C14—C18	1.383 (3)
C3—C4	1.381 (3)	C14—C15	1.385 (3)
C3—H3	0.9300	C15—C16	1.381 (3)
C4—C5	1.384 (3)	C15—H15	0.9300
C4—C6	1.509 (3)	C16—C17	1.373 (3)
C5—N1	1.354 (2)	C16—H16	0.9300
C5—H5	0.9300	C17—N2	1.351 (2)
C6—O1	1.412 (2)	C17—H17	0.9300
C6—H6A	0.9700	C18—N2	1.334 (2)
C6—H6B	0.9700	C18—H18	0.9300
C7—O1	1.380 (2)	Cu1—N2^i^	2.0153 (16)
C7—C12	1.383 (3)	Cu1—N2^ii^	2.0153 (16)
C7—C8	1.386 (3)	Cu1—N1	2.0669 (16)
C8—C9	1.389 (3)	Cu1—N1^iii^	2.0669 (16)
C8—H8	0.9300	Cu1—O3	2.5460 (15)
C9—C10	1.383 (3)	N2—Cu1^iv^	2.0153 (16)
C9—H9	0.9300	N3—O4	1.226 (7)
C10—O2	1.380 (2)	N3—O5	1.229 (2)
C10—C11	1.382 (3)	N3—O4'	1.253 (8)
C11—C12	1.389 (3)	N3—O3	1.259 (2)
C11—H11	0.9300	O4—O4'	0.773 (8)
C12—H12	0.9300		
			
N1—C1—C2	122.43 (18)	H13A—C13—H13B	108.7
N1—C1—H1	118.8	C18—C14—C15	117.88 (17)
C2—C1—H1	118.8	C18—C14—C13	118.12 (17)
C1—C2—C3	119.7 (2)	C15—C14—C13	124.00 (18)
C1—C2—H2	120.1	C16—C15—C14	118.58 (19)
C3—C2—H2	120.1	C16—C15—H15	120.7
C2—C3—C4	118.72 (18)	C14—C15—H15	120.7
C2—C3—H3	120.6	C17—C16—C15	120.29 (18)
C4—C3—H3	120.6	C17—C16—H16	119.9
C3—C4—C5	118.58 (17)	C15—C16—H16	119.9
C3—C4—C6	122.69 (17)	N2—C17—C16	121.55 (17)
C5—C4—C6	118.65 (18)	N2—C17—H17	119.2
N1—C5—C4	123.05 (19)	C16—C17—H17	119.2
N1—C5—H5	118.5	N2—C18—C14	123.85 (17)
C4—C5—H5	118.5	N2—C18—H18	118.1
O1—C6—C4	107.81 (17)	C14—C18—H18	118.1
O1—C6—H6A	110.1	N2^i^—Cu1—N2^ii^	180.000 (1)
C4—C6—H6A	110.1	N2^i^—Cu1—N1	89.35 (6)
O1—C6—H6B	110.1	N2^ii^—Cu1—N1	90.65 (6)
C4—C6—H6B	110.1	N2^i^—Cu1—N1^iii^	90.65 (6)
H6A—C6—H6B	108.5	N2^ii^—Cu1—N1^iii^	89.35 (6)
O1—C7—C12	124.94 (18)	N1—Cu1—N1^iii^	180.000 (1)
O1—C7—C8	115.08 (18)	N2^i^—Cu1—O3	86.22 (6)
C12—C7—C8	119.98 (17)	N2^ii^—Cu1—O3	93.78 (6)
C7—C8—C9	120.21 (19)	N1—Cu1—O3	92.59 (6)
C7—C8—H8	119.9	N1^iii^—Cu1—O3	87.41 (6)
C9—C8—H8	119.9	C1—N1—C5	117.48 (16)
C10—C9—C8	119.67 (18)	C1—N1—Cu1	118.31 (12)
C10—C9—H9	120.2	C5—N1—Cu1	123.96 (13)
C8—C9—H9	120.2	C18—N2—C17	117.81 (16)
O2—C10—C11	114.98 (18)	C18—N2—Cu1^iv^	118.98 (12)
O2—C10—C9	124.83 (18)	C17—N2—Cu1^iv^	123.21 (13)
C11—C10—C9	120.18 (17)	O4—N3—O5	118.1 (5)
C10—C11—C12	120.20 (19)	O4—N3—O4'	36.3 (4)
C10—C11—H11	119.9	O5—N3—O4'	118.6 (4)
C12—C11—H11	119.9	O4—N3—O3	119.1 (5)
C7—C12—C11	119.76 (19)	O5—N3—O3	120.22 (18)
C7—C12—H12	120.1	O4'—N3—O3	117.2 (5)
C11—C12—H12	120.1	C7—O1—C6	117.53 (16)
O2—C13—C14	106.10 (16)	C10—O2—C13	117.89 (16)
O2—C13—H13A	110.5	N3—O3—Cu1	134.44 (13)
C14—C13—H13A	110.5	O4'—O4—N3	73.8 (10)
O2—C13—H13B	110.5	O4—O4'—N3	69.9 (10)
C14—C13—H13B	110.5		

Symmetry codes: (i) −*x*+1, −*y*+2, −*z*+1; (ii) *x*−2, *y*, *z*−1; (iii) −*x*−1, −*y*+2, −*z*; (iv) *x*+2, *y*, *z*+1.
